# Experimental Granulomatous Amebic Encephalitis Caused by *Acanthamoeba castellanii*

**DOI:** 10.3390/tropicalmed9070145

**Published:** 2024-06-28

**Authors:** Samuel da Luz Borges, Eberson da Silva de Macedo, Felipe Alexandre Vinagre da Silva, Brenda Jaqueline de Azevedo Ataíde, Nívia de Souza Franco Mendes, Adelaide da Conceição Fonseca Passos, Suellen Alessandra Soares de Moraes, Anderson Manoel Herculano, Karen Renata Herculano Matos Oliveira, Carlomagno Pacheco Bahia, Silvio Santana Dolabella, Evander de Jesus Oliveira Batista

**Affiliations:** 1 Laboratório de Protozoologia, Núcleo de Medicina Tropical, Universidade Federal do Pará, Belém 66055-240, Brazil; eberson.macedo@icb.ufpa.br (E.d.S.d.M.); felipe.vinagre.silva@icb.ufpa (F.A.V.d.S.); evander@ufpa.br (E.d.J.O.B.); 2 Laboratório de Biologia, Campus Castanhal, Instituto Federal de Educação, Ciência e Tecnologia do Pará, Castanhal 68740-970, Brazil; 3 Laboratório de Neurofarmacologia Experimental, Instituto de Ciências Biológicas, Universidade Federal do Pará, Belém 66075-110, Brazil; brenda.efo@hotmail.com (B.J.d.A.A.); nivia.mendes@ics.ufpa.br (N.d.S.F.M.); adelaide.passos@gmail.com (A.d.C.F.P.); suellen@ufpa.br (S.A.S.d.M.); herculano@ufpa.br (A.M.H.); karen@ufpa.br (K.R.H.M.O.); 4 Laboratório de Neuroplasticidade, Instituto de Ciências da Saúde, Universidade Federal do Pará, Belém 66055-240, Brazil; bahiacp@ufpa.br; 5 Laboratório de Entomologia e Parasitologia Tropical, Universidade Federal de Sergipe, Aracajú 49060-108, Brazil; dolabella@ufs.br

**Keywords:** *Acanthamoeba*, brain, diagnostic, experimental infection, encephalitis, rat

## Abstract

Acanthamoeba genus can affect humans with diseases such as granulomatous amebic encephalitis (GAE), a highly lethal neuroinfection. Several aspects of the disease still need to be elucidated. Animal models of GAE have advanced our knowledge of the disease. This work tested Wistar rats (*Rattus norvegicus albinus*) as an animal model of GAE. For this, 32 animals were infected with 1 × 10^6^ *A. castellanii* trophozoites of the T4 genotype. Ameba recovery tests were carried out using agar plates, vascular extravasation assays, behavioral tests, and histopathological technique with H/E staining. Data were subjected to linear regression analysis, one-way ANOVA, and Tukey’s test, performed in the GraphPad Prism^®^ 8.0 program, with a significance level of *p* < 0.05. The results revealed the efficiency of the model. Amebae were recovered from the liver, lungs, and brain of infected animals, and there were significant encephalic vascular extravasations and behavioral changes in these animals, but not in the control animals. However, not all infected animals showed positive histopathology for the analyzed organs. Nervous tissues were the least affected, demonstrating the role of the BBB in the defense of the CNS. Supported by the demonstrated evidence, we confirm the difficulties and the feasibilities of using rats as an animal model of GAE.

## 1. Introduction

The genus *Acanthamoeba* is free-living, inhabiting practically all natural environments [1]. The ubiquity of *Acanthamoeba* represents a significant threat to human health, as they can be highly pathogenic, affecting humans with diseases such as cutaneous acanthamoebiasis (CA) whose lesions include nodules, ulcers, bedsores, and abscesses, often appearing on the face and extremities [2]. Clinical studies also describe a severe corneal infection called *Acanthamoeba* keratitis (AK) which is closely associated with the use of contact lenses [3].

At the central nervous system level, *Acanthamoeba* is able to induce a severe disease called granulomatous amebic encephalitis (GAE) which causes a lethal infection in the brain. The neurological symptoms associated with this central nervous system infection include an altered mental state, seizures, focal neurological signs, ataxia, lethargy, neck stiffness, and personality changes, and they can lead to death due to increased intracranial pressure [4]. Most of these infections are opportunistic and affect immunosuppressed individuals such as patients using corticosteroids, those developing autoimmune diseases or immunosuppressive infections such as HIV/AIDS, and organ-transplanted individuals [5]. In these patients there is a change in the status of the infection and the host–pathogen relationship, developing from an asymptomatic or oligosymptomatic form to a disseminated form that affects several organs and can lead to the death of the host [6].

Among the diseases caused by *Acanthamoeba* spp., we highlight GAE which, despite an increased number of diagnosed cases and poor prognosis resulting in the death of approximately 95% of patients, is neglected and in need of more research and attention. Some aspects of the disease, such as the pathogenesis and immune response, still need to be elucidated [7].

Much of the research that seeks to elucidate these aspects of GAE is carried out in vitro and the conclusions cannot always be extrapolated to clinical situations. Studies using animal models for experimental infections, which mimic the natural processes of contagion, dissemination, and infection more closely [8], have traditionally been conducted using mice [9], while rats and other laboratory animals are rarely used.

As with other pathologies, the use of animal models to analyze the course of GAE is essential to advance our knowledge of key aspects, such as pathogenesis and immune response, which is necessary for improving the diagnosis and treatment of the disease. To this end, the main aim of the current study was to characterize an animal model of GAE utilizing Wistar rats (*Rattus norvegicus albinus*) infected via intranasal instillation with a pathogenic strain of Acanthamoeba castellanii, with a focus on alterations in the brain, liver, and lung evoked by infection.

## 2. Materials and Methods

### 2.1. Ethical Issues

This research was approved by the Animal Use Ethics Committee of the Universidade Federal do Pará (protocol number 3980160818), following all the norms and guidelines of the National Council for the Control of Animal Experimentation.

### 2.2. Amebae and Reactivation of Strain Virulence

The amebae used were of the *A. castellanii* species (ATCC 50492) obtained from a clinical case of human keratitis and previously identified as the T4 genotype, which is very involved in cases of GAE [10]. The cells were kept for a long time in PYG (peptone–yeast–glucose) medium until their virulence was reactivated after passage via healthy Wistar rats, in which they were able to cause pulmonary infection. Once they were rescued from the lungs after maceration of the tissue, reactivated amebae (now called T4r) were used to infect the experimental animals.

### 2.3. Cultivation and Maintenance of Amebae for Infection

T4r amebae were maintained and cultured in Falcon tubes containing PYG, with replications being performed every 7 days. On the days when the experimental infections were carried out, the number of cells in culture was adjusted after counting in the Neubauer chamber in order to obtain 1 × 10^6^ trophozoites/mL of medium to be used for the inoculation of each animal.

### 2.4. Animal Model

The animal model of GAE involved 64 healthy Wistar male rats, aged two to three months with a body mass in the range of 250–300 g, from the Central Animal Facility of the Institute of Biological Sciences of the Federal University of Pará (ICB-UFPA). The animals were analyzed to detect any deformities, treated against ectoparasites, and then placed in standard polypropylene cages in a laboratory environment with a 12 h light/dark cycle, receiving water and food ad libitum.

### 2.5. Experimental Groups

The four experimental animal groups (G1–G4) contained 16 animals each. The G1 group was used to evaluate the infection route efficiency by recovering amebae from organ tissues after culture in non-nutrient agar, the G2 group was used to evaluate the integrity of the blood–brain barrier (BBB) and vascular extravasation, the G3 group was used for behavioral analysis, and the G4 group for histological analysis. In each group, 8 animals were immunosuppressed and infected via intranasal inoculation with 1 × 10^6^/mL T4r trophozoites, and 8 animals were used as a control group; 4 were immunosuppressed and 4 were not.

### 2.6. Immunosuppression and Induction of Experimental Infection

The animals in each of the experimental groups G1, G2, G3, and G4 underwent immunosuppression via intraperitoneal injection of 1 daily dose of dexamethasone (5 mg/kg) for 3 consecutive days. One day after the last dose of the immunosuppressant, the mice were inoculated intranasally with 200 µL of a PYG solution containing 1 × 10^6^/mL T4r trophozoites [11]. The animals used as controls were intranasally instilled with 200 µL of the sterile PYG medium, and half (4) were also immunosuppressed while the other half were not. 

### 2.7. Recovery of Amebae from Non-Nutrient Agar

After 30 days of infection, the animals in group G1 were anesthetized via intraperitoneal injection with a combination of ketamine hydrochloride (100 mg/kg) and xylazine hydrochloride (5.0 mg/kg), and the “tail test” used to verify an adequate state of analgesia and sedation to proceed to a surgical plan [12]. The animals were then euthanized, and the brain, lungs, and liver were extracted from each animal. After macroscopic analysis, the organs were macerated separately, and some of the resulting material was dripped onto Petri dishes containing non-nutrient agar covered with heat-inactivated *Escherichia coli* cells. The Petri dishes were incubated at room temperature for later direct analysis under a magnifying glass and via the mounting of slides for microscopic analysis.

### 2.8. Vascular Permeability Test

The integrity of the BBB after T4r infection was evaluated using the vascular permeability test via the intracardiac injection of a sterile 2.0% solution of Evans blue dye in distilled water [13].

The animals in the G2 group used for this procedure were anesthetized and opened to expose the heart for injection with 500 µL of a 2% solution of Evans blue dye [14]. Dye injection was performed manually using a 1 mL syringe. The removal of blood and excess dye from the animal’s blood vessels was performed as previously described by Nag [15]. The brains were then removed and placed in a Petri dish to assess the presence of vascular extravasation in the animal’s encephalic microvasculature, performed firstly with the naked eye and then under a magnifying glass to capture images. Quantification of extravasated dye was performed as described by Ataíde et al. [16].

### 2.9. Behavioral Test

The rapid murine coma and behavior scale (RMCBS) was used to assess locomotor and exploratory activities, in addition to health status and the course of neural infection by T4r in rats from the G3 group. The tests were performed as previously described by Carrol et al. [17], including all 10 parameters recommended by the RMCBS.

### 2.10. Histopathology

After 30 days of infection, the animals in the G4 group were anesthetized and opened to expose the heart for injection with PBS, as described above. Then, 300 mL of 4% paraformaldehyde (PFA) diluted in 0.9% PBS was perfused for tissue fixation, as verified by the inoculated volume and the rigidity of the head and upper limbs of the animal [18]. Then, the brains were removed and placed in Falcon tubes with 4% PFA for 24 h, before being embedded in paraffin, processed, and transported to microtomy to obtain 4 µm sections, which were mounted on slides and stained with hematoxylin and eosin (H/E).

### 2.11. Statistical Analysis

The vascular extravasation quantification data were subjected to linear regression analysis to obtain the correlation coefficient (r) between optical densities and Evans blue concentrations [19].

One-way ANOVA and Tukey’s tests were also applied to verify the distribution and statistical differences between the means of the control and experimental groups. The tests were performed using the GraphPad Prism^®^ 8.0 program and the significance level was *p* < 0.05.

## 3. Results

### 3.1. Recovery of Amebae from Non-Nutrient Agar

**Figure 1:**
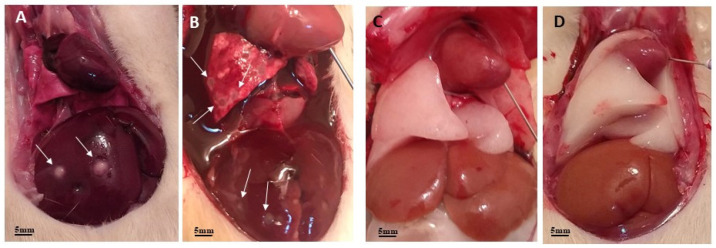
Anatomopathological analysis of the liver and lungs of experimental animals. Images (**A**,**B**) show several liver and lung lesions (arrows) compatible with T4r infection. Images (**C**,**D**) show these organs preserved in control animals.

After 30 days of intranasal infection with 1 × 10^6^ T4r cells, the animals in the G1 group were euthanized and the brain, lungs, and liver were extracted from each animal. The anatomopathological analysis revealed liver and lung lesions (Figure 1) compatible with infection by *A. castellanii* T4r.

**Figure 2:**
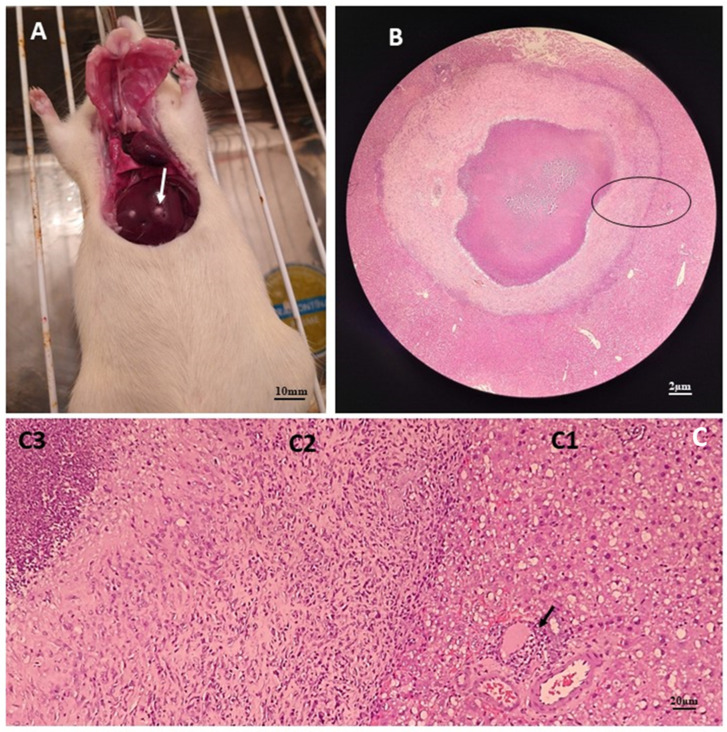
Anatomopathological and histopathological images of the liver of an infected rat. Image (**A**) shows an animal undergoing surgery with liver lesions (arrow) compatible with *A. castellanii* infection. Image (**B**) shows a histological section with an overview of one of the liver lesions showing three well-defined areas (circle), at a final magnification of 32×. Image (**C**) shows these three areas divided into (**C1**) which represents the peripheral area and shows acidophilic hepatocytes with hyperchromatic nuclei, in addition to mild steatosis. The arrow points to an inflammatory focus. (**C2**) is the intermediate area characterized by a halo of fibrosis, and (**C3**) represents the central area of the lesion, characterized by the presence of necrosis with a large number of degenerated neutrophils, in a histological section after H/E staining with a final magnification of 200×.

One of the liver lesions was sectioned and underwent a histopathological procedure with H/E staining, revealing the particularities of this lesion (Figure 2).

**Figure 3:**
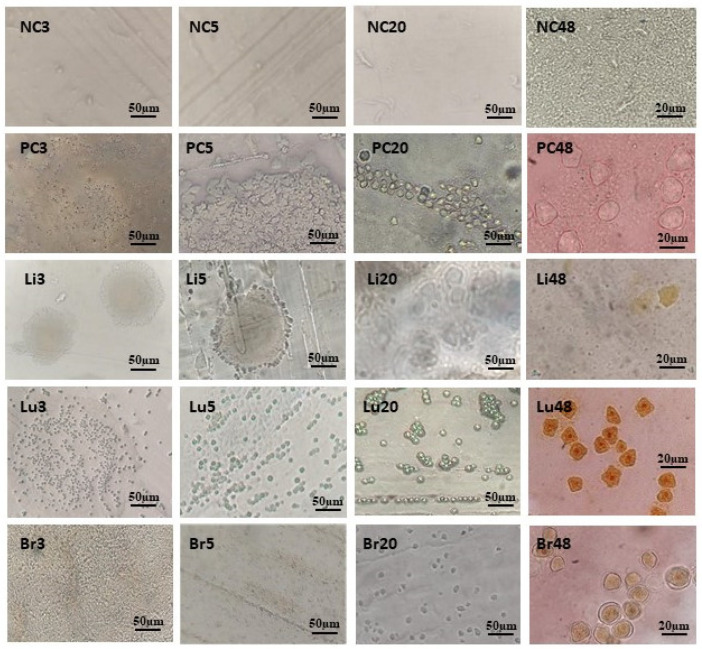
Photomicrographs of plates and slides mounted with material from amoeba culture in agar. (NC): Negative control plates show no amebae. (PC): Positive control plates containing T4r cells maintained in PYG show; in PC3, multiple trophozoites at the culture addition site; in PC5, trophozoites in the process of encysting; in PC20, cysts formed in the trail of *E. coli*; and in PC48, there were cysts characteristic of A. castellanii using optical microscopy (OM) with a final magnification of 400×. (Li): Here, the plate was seeded with liver material from a T4r-infected animal. Li3 and Li5 show the site of addition of liver material; and Li20 and Li48 show T4r cysts in a slide visualized using OM with a final magnification of 400×. (Lu): Here, the agar plate was seeded with lung material from an infected animal; Lu3 shows lung addition site; Lu5 shows cysts in *E. coli* tracks; Lu20 shows numerous mature cysts; and Lu48 shows cysts characteristic of *A. castellanii* in a slide stained with Lugol’s solution and visualized using OM with a final magnification of 400×. (Br): Here, the plate was seeded with encephalic material from the brain of an infected animal. Br3 shows the site of material addition; Br5 shows *E. coli* trail; Br20 shows several trophozoites and cysts; and Br48 shows a cyst characteristic of *A. castellanii* in a slide stained with Lugol’s solution and visualized using OM with a final magnification of 400×.

After analysis and photographic recording, the organs were macerated and the resulting material was dripped onto Petri dishes containing non-nutrient agar covered with heat-inactivated *E. coli* cells, establishing an ameba culture. The plates were photographed, and aliquots were taken to mount slides for microscopy. The images in Figure 3 show samples of the negative control (NC—only PYG on the agar plate), the positive control (PC—amebae in PYG on the agar plate), liver (Li—liver macerate on agar plate), lung (Lu—lung macerate on agar plate), and brain (Br—brain macerate on agar plate), on days 3, 5, 20, and 48 after establishing the culture. Image analysis revealed rapid growth of amebae on plates with lung maceration, and slower growth of amebae on plates with liver and neural maceration.

Plates containing agar with macerated animal organs and cysts remained viable at room temperature for up to 110 days after plating, after which the drying of the material caused many cracks to form in the middle.

**Figure 4:**
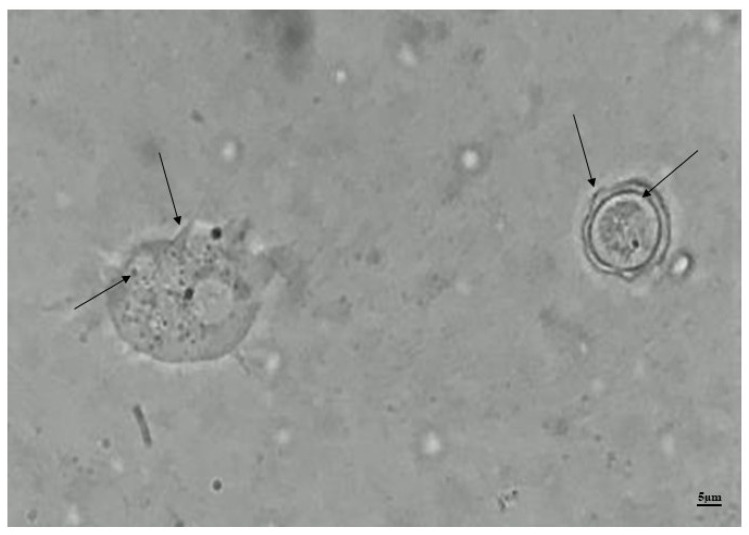
Photomicrograph of a trophozoite and cyst of *A. castellanii* in PYG. On the left in the image we have a trophozoite showing acanthopodia and contractile vacuoles, and on the right a cyst there is a showing ecto- and endocysts (indicated by arrows), visualized using optical microscopy with a final magnification of 1000×.

T4r cells were maintained in PYG medium prior to carrying out the experimental infections. Figure 4 shows an image of trophozoites and cysts characteristic of *A. castellanii* genotype T4, placed together on a freshly prepared slide using the maintenance medium from these cells.

### 3.2. Vascular Permeability Test

**Figure 5:**
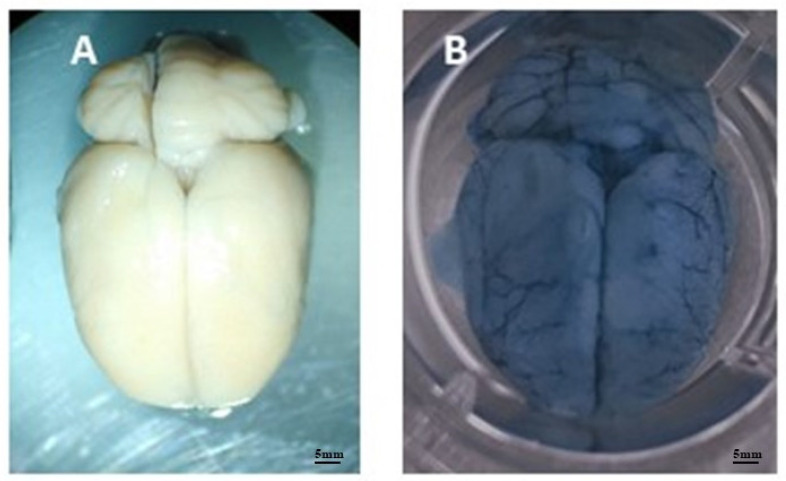
Photographs of the encephalic vasculature of an infected and non-infected animal. Image (**A**) shows the brain of a control rat without signs of vascular leakage. Image (**B**) shows a rat brain 8 weeks after infection, showing significant leakage of Evans blue dye.

**Figure 6:**
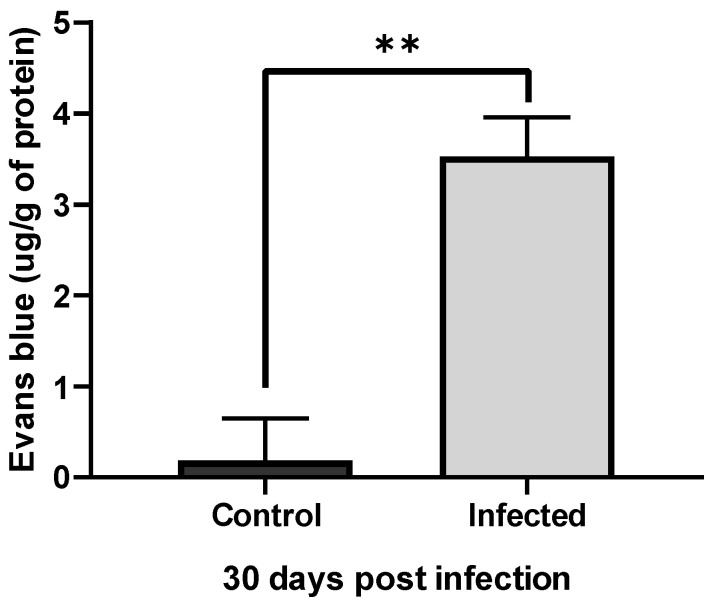
Graph with vascular extravasation results from control and infected animals. Comparison of the mean quantification of extravasated dye in the brain of control rats versus rats infected with T4r shows a significant difference. Data are presented as follows: ** (*p <* 0.01).

The integrity of the BBB after infection by *A. castellanii* was assessed by direct analysis of the brain microvasculature of animals in group G2 (Figure 5) and by quantification of Evans blue dye extracted from brains stored in formamide, performed using an ELISA reader. Extravasated Evans blue was expressed as mg dye/g tissue (Figure 6).

### 3.3. Behavioral Tests

**Figure 7:**
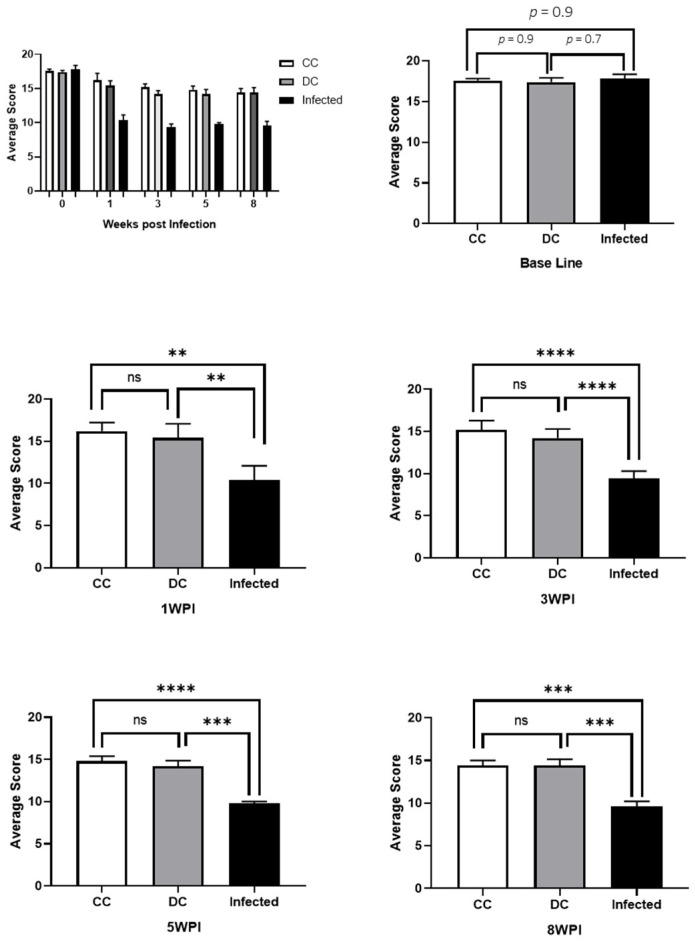
Image with comparative graphs of the results of the RMCBS behavioral test in control and infected rats. (CC) Clean control rats; (DC) control rats with application of dexamethasone; (Infected) rats infected with T4r. There were no statistical differences between any of the 3 groups at baseline (week 0). There were no statistical differences between CC and DC in any of the tests. There was a statistical difference between control animals (CC and DC) and infected animals in all post-infection tests, with the smallest difference observed at 1WPI (*p* < 0.01) and the largest at 3WPI (*p* < 0.0001). WPI = week post infection. Data are presented as follows: ns (no significant difference), ** (*p* < 0.01), *** (*p* < 0.001), **** (*p* < 0.0001).

The RMCBS test was used to investigate locomotor and exploratory activities, and anxiety levels in animals in response to neural infection with T4r. The animals were evaluated at week 0, 1, 3, 5, and 8 post-infection. Figure 7 summarizes the results obtained from the RMCBS.

### 3.4. Histopathology

After 30 days of incubation, the animals in the G4 group were perfused, and the brain, lungs, and liver fixed in 4% PFA and embedded in paraffin for mounting and staining onto glass slides ready for viewing under an optical microscope. Ten slides stained with H/E were mounted and analyzed for each perfused organ.

**Figure 8:**
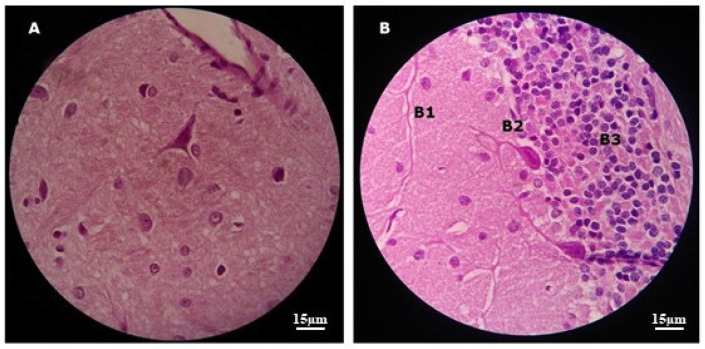
Images showing histopathological sections of the brain of a control animal after H/E staining: In (**A**) we can see the cerebral cortex with preserved tissue architecture. In (**B**) is the cerebellum with tissue preservation in the molecular (**B1**), Purkinje (**B2**), and granulosa (**B3**) layers. Original 1000× magnification using MO.

Histopathological analysis of brains from infected animals did not detect T4r cysts or trophozoites. Control animals showed tissue preservation in the brain regions analyzed (Figure 8).

## 4. Discussion

Since the first experiments with animal models of neural infection by *Acanthamoeba* were carried out by Culbertson et al. [20] using mice and monkeys, many discoveries have been made while new questions have arisen. Some aspects, relating mainly to pathogenesis, immune response, and treatment, remain unclear.

After the first confirmed reports in the 1970s [21,22], cases of GAE were reported on all continents [23] and the disease continues to have a very poor prognosis. According to Visvesvara et al. [24], the high mortality that accompanies the poor prognosis in GAE is largely due to the lack of knowledge about amebic diseases. The situation does not seem to have changed in recent years. According to Kot et al. [25], no definitive protocol for GAE treatment has yet been established. The lack of trained professionals and protocols for etiological recognition are at the heart of this problem, which can lead to significant underreporting and errors in the diagnosis of this disease [26]. Therefore, the search for diagnostic tools and protocols for GAE is essential to improving its poor prognosis [27]. In this context, the use of animal models is essential in order to support the construction of these tools and protocols.

In the present work, various tests were used to evaluate Wistar rats as a viable animal model of GAE. 

### 4.1. Recovery of Amebae from Non-Nutrient Agar

The *Acanthamoeba castellanii* T4r cells used for the intranasal infection of experimental rats caused systemic infection, with amebae being recovered from the brain, lungs, and liver of the animals. These results agree with Veríssimo et al. [11] and Omaña-molina et al. [9], who achieved similar results with *A. castellanii* seeded on non-nutrient agar plates.

It is worth mentioning that the growth of *A. castellanii* occurred on both non-nutrient agar plates whose nutritional source for the amebae were bacteria inactivated by heat, as other researchers have previously reported [28,29,30], and on plates containing sterile PYG as the only nutritional source. This is interesting since the methodology using PYG as a nutritional source alone is simpler and less expensive, which may be useful when bacterial strains are not available. Furthermore, growth in medium with bacterial strains may be slower [31].

It is also important to point out that the animals used as controls in all experiments survived the entire period of tests, and post-mortem examinations did not reveal any damage to the internal organs, nor were amebae recovered from the organs of these animals.

### 4.2. Vascular Permeability Test

The images of the brain microvasculature of the animals and the quantification of the Evans blue dye extracted from the brains confirmed the efficiency of the model of intranasal infection of Wistar rats by *Acanthamoeba castellanii*, as well as the hematogenous dissemination and the ability of the ameba to cross the blood–brain barrier of the host, as reported by Khan and Siddiqui [32]. Neural infections by these amebae in humans generally occur in the lower respiratory tract, suggesting that the invasion of the CNS is via the olfactory neuroepithelium, as was also observed by Kuhlencord et al. [33] and Omaña-molina et al. [9], or at the site of the BBB, in accordance with findings by Sissons et al. [34] and Khan [35].

Passage through the BBB, despite depending on enzymatic processes and the contact between amebae and brain microvascular endothelial cells (BMEC), is facilitated in part by the exacerbated immune response of the host. According to Baig [36], in addition to violating the BBB, this immune system response is the main cause of brain damage in the course of GAE, in addition to the action of toxins and enzymes secreted by the ameba [37].

**Figure 9:**
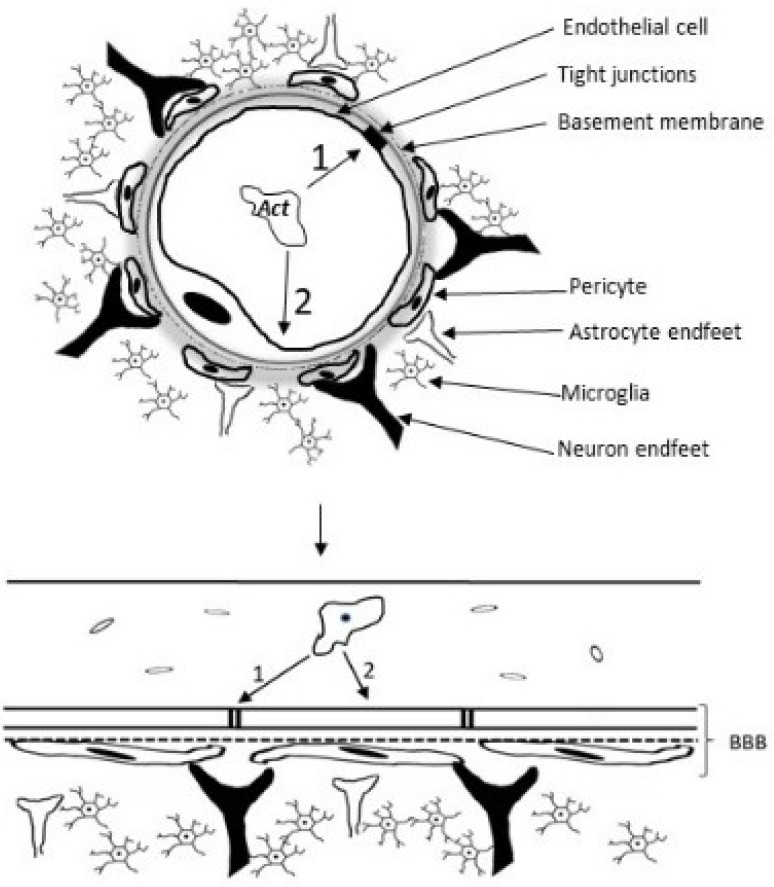
Schematic diagram of a neurovascular unit showing the elements of the blood–brain barrier and the passageways of Acanthamoeba (Act) into the brain parenchyma. 1—Paracellular way: Tight junction elements such as occludin and ZO-1 protein are degraded by *Acanthamoeba* proteases, in a contact-independent mechanism. (Note: serine proteases, metalloproteases and ecto-ATPases facilitate transmigration and passage to deeper regions of the brain). 2—Transcellular pathway: mannose binding protein (MBP) binds to brain microvascular endothelial cell receptors, altering the cycle and causing its death, in a contact-dependent mechanism.

*Acanthamoeba* spp. degrades occludin and ZO-1 protein, fundamental components of tight junctions and endothelial cell selectivity, indicating the use of the paracellular route as an important strategy to access the CNS (Figure 9). This route involves the crossing of the BBB between endothelial cells and is used by other protozoans such as *Plasmodium falciprum*, *Trypanosoma* spp. and *Toxoplasma gondii* [38,39]. *Acanthamoeba* spp. also use the transcellular route, where they paralyze the BMEC cell cycle resulting in necrosis or apoptosis and loss of BBB integrity [40].

Control animals did not show significant vascular extravasation or brains with microvasculature altered by the dye, as shown in the images.

It should be noted that infected animals had inflammation and necrosis of the nostrils and olfactory bulb, indicating invasion of the brain via the nasal mucosa and olfactory nerves, as has been demonstrated in other studies with experimental GAE [41,42].

### 4.3. Behavioral Tests

During the intimate relationship established between parasites and their host animals, detectable behavioral changes may occur in the host. These alterations are assessed by the animals’ performance in behavioral tests that measure the final manifestations of neural functions, such as motor coordination, memory, or states of anxiety [43]. In our research, we used the RMCBS test, an objective and quantitative scale created for use with mice, seeking to compare the results obtained with the findings of the vascular permeability test in response to neural infection of the animals with *A. castellanii* T4r.

In agreement with the findings of vascular permeability assays, which demonstrated disruption of the blood–brain barrier and cerebral vascular extravasation, the RMCBS showed a significant difference (*p* < 0.01) in the mean score of control rats compared to infected animals in all tests post infection.

The mechanisms that induce behavioral changes in parasitized animals are not completely known [44]. Side effects of damage to the nervous system, such as altered cytokine expression of IFN-γ, TNF, IL-2 and IL-12 [45], or the action of substances released by parasites that act as neurotransmitters influencing host behavior may be involved [46].

Certainly, these behavioral parameters are fundamental in determining, together with other assays, the presence of parasites in the CNS, as well as the presence and extent of lesions resulting from parasitic invasion.

### 4.4. Histopathology

Not all infected animals showed positive histopathology in the analyzed organs. Nervous tissue was the least affected, demonstrating the clear role of the BBB in defense of the CNS, as described by Daneman and Prat [47]. In the work by Markowitz et al. [48], histological changes in infected mice were minimal in several organs, including 9/10 animals with unchanged brains even 35 days after infection by *A. castellanii*.

To increase the chances of detection of T4r by histopathology of the analyzed organs, especially the brain, the infection period was long, as recommended by Ramirez et al. [49]. However, this did not guarantee 100% detection of lesions, cysts, or trophozoites consistent with the histopathology of *A. castellanii* infection. This highlights the importance of molecular studies (not carried out in this study) using PCR to detect the presence of *Acanthamoeba* in tissue samples from infected animals, as performed by Gianinazzi et al. [42].

## 5. Conclusions

The presented results show that Wistar rats represent an efficient animal model for studies related to GAE. As evidenced in our results, after infection with *A. castellanii* the animals presented evidenced alterations in the brain, liver, and lungs like those observed in histopathological studies performed in human patients. However, some aspects of animal infections still represent a challenge, since some infected animals did not show behaviors or histopathology typical of T4r infection, indicating the need for further research aimed at expanding model tests and including analysis of the host immune response, in the search for answers to the questions that still exist, helping to advance knowledge and combat this pathology.

## Data Availability

The data presented in this study are available in this article and on request from the corresponding author.
